# Short-term arm immobilization reduces maximal force and alters fatigue responses to a relative-intensity task in team-sport athletes: a randomized controlled trial

**DOI:** 10.3389/fphys.2026.1977296

**Published:** 2026-10-28

**Authors:** Siwar Erriahi, Wael Daab, Achraf Ammar, Firas Zghal, Sirine Hamdi, Ines Ben Hsen, Mohamed Amine Bouzid

**Affiliations:** 1 Research Laboratory: Education, Motricity, Sport and Health, EM2S, LR19JS01, High Institute of Sport and Physical Education, University of Sfax, Sfax, Tunisia; 2 College of Sport Science, University of Kalba, Sharjah, United Arab Emirates; 3 Department of Training and Movement Science, Institute of Sport Science, Johannes Gutenberg-University Mainz, Mainz, Germany; 4 Research Laboratory, Molecular Bases of Human Pathology, LR19ES13, Faculty of Medicine of Sfax, University of Sfax, Sfax, Tunisia; 5 Department of Nutrition and Food Technology, School of Agriculture, The University of Jordan, Amman, Jordan; 6 High Institute of Sport and Physical Education of Sfax, University of Sfax, Sfax, Tunisia; 7 Complexité, Innovation, Activités Motrices et Sportives (CIAMS), Université Paris-Saclay, Orsay, France

**Keywords:** immobilization, muscle disuse, neuromuscular fatigue, peripheral fatigue, voluntary activation

## Abstract

**Background:**

Short-term limb immobilization induces rapid losses in muscle size and strength, but its effects on performance fatigability and the underlying central and peripheral neuromuscular mechanisms remain unclear. This study examined the effects of three weeks of non-dominant arm immobilization on neuromuscular function and exercise-induced fatigability in team-sport athletes.

**Methods:**

In a parallel-group randomized controlled trial, 24 team-sport athletes were randomly allocated to an immobilization group or a control group. Twelve team-sport athletes underwent three weeks of non-dominant arm immobilization (IMMO), while twelve served as controls (CG). Before and after the intervention, participants performed a sustained isometric contraction at 50% of maximal voluntary contraction (MVC) of the elbow flexors until task failure. Because the target force was recalculated as 50% of the MVC measured at each visit, the absolute target force was lower after immobilization in the IMMO group. Biceps brachii activation was assessed by electromyography (EMG), while central and peripheral components were quantified using voluntary activation (VA), potentiated twitch force (Q_tw,pot_), and M-wave responses. Upper-arm muscle-and-bone area (MBA) was estimated anthropometrically.

**Results:**

In the IMMO group, immobilization reduced anthropometrically estimated MBA (−7.3%) and pre-exercise MVC (−22.9%), whereas neither changed in the control group. Consequently, the absolute target force of the fatiguing task was lower after immobilization in the IMMO group (89.9 ± 17.5 N before vs 69.3 ± 11.9 N after). Under this lower absolute demand, endurance time increased (220.88 ± 102.7 vs 281.65 ± 99.65 s, p < 0.001) and the exercise-induced decline in MVC was smaller (−31.5 ± 10.2% before vs −14.4 ± 7.8% after immobilization; group × condition × exercise interaction, F(1,22) = 15.74, p < 0.001, η_p_² = 0.42, 95% CI 0.10–0.62). The exercise-induced decline in Q_tw,pot_ was also smaller after immobilization in the IMMO group (−38.9% before vs −15.2% after; interaction F(1,22) = 4.58, p = 0.044, η_p_² = 0.17, 95% CI 0.00–0.42), whereas it remained large in the control group. Voluntary activation (VA) and RMS/M_max_ decreased after exercise, but no group × condition × exercise interaction was detected for VA (p = 0.130) or RMS/M_max_ (p = 0.391). Given the sample size, these non-significant interactions indicate that a differential central or sarcolemmal response was not detected, not that it was absent.

**Conclusions:**

In conclusion, short-term immobilization impaired maximal force-generating capacity while increasing endurance time during a sustained submaximal task performed at the same relative, but a lower absolute, intensity. The exercise-induced declines in both maximal force and potentiated twitch force were attenuated after immobilization, whereas no differential change in voluntary activation or M-wave responses was detected. Because this peripheral signature occurred under a lower absolute load, it most plausibly reflects a smaller peripheral fatigue response to a reduced absolute demand rather than an intrinsic enhancement of contractile fatigue resistance. These findings describe altered fatigability during a relative-intensity task and should not be interpreted as improved intrinsic fatigue resistance.

**Clinical trial registration:**

https://pactr.samrc.ac.za/, identifier PACTR202607531230552.

## Introduction

Limb immobilization is commonly prescribed following musculoskeletal injury or surgical intervention to facilitate tissue healing by minimizing deleterious mechanical stress, such as excessive joint motion or muscle lengthening (Kannus and Järvinen, 1990; Leigh et al., 1999; Stevenson et al., 2003; Singer and Friedrich, 2023). Despite its clinical necessity, immobilization, even when applied for short durations, induces rapid and pronounced alterations in the neuromuscular system. These alterations include reductions in muscle strength, muscle atrophy, impairments in neuromuscular function, and decrements in physical performance, all of which may compromise rehabilitation outcomes. Such effects are particularly problematic in athletic populations, where maintaining neuromuscular performance and ensuring a timely return to sport are of paramount importance.

One key functional consequence of immobilization that remains insufficiently characterized is performance fatigability, defined as a task-dependent decline in a muscle or muscle group’s ability to sustain a prescribed force or power output over time, or to complete a given amount of work under standardized conditions (Edwards, 1981; Fitts, 1994; Enoka and Duchateau, 2016). Previous studies examining the effects of muscle disuse, such as bed rest, cast immobilization, or paralysis, on performance fatigability have yielded inconsistent findings. While several investigations report increased fatigability following disuse (Rochester et al., 1995; Shaffer et al., 2000; Miles et al., 2005; Mulder et al., 2007), others observe no change (Cameron and Calancie, 1995; Kamiya et al., 2000; Seki et al., 2001; Kamiya et al., 2004; Andrushko et al., 2018) or even improvements in fatigue resistance (Yue et al., 1997; Semmler et al., 2000; Deschenes et al., 2002; Clark et al., 2008). These observations appear paradoxical given the well-documented structural and metabolic adaptations associated with disuse-induced muscle atrophy, including a shift toward faster myosin heavy chain isoforms and a greater reliance on glycolytic metabolism, adaptations typically linked to reduced fatigue resistance (Fitts et al., 2000; Stein and Wade, 2005). Additionally, the reduction in absolute force-generating capacity associated with muscle atrophy may indirectly attenuate performance fatigability by lowering intramuscular pressure and improving the balance between muscle perfusion and metabolic demand (Semmler et al., 2000; Bodor, 2002).

Performance fatigability depends on the contractile capabilities of the involved muscles and the capacity of the nervous system to provide an adequate neural drive (Enoka and Duchateau, 2016). According to contemporary conceptual models, performance fatigability arises from alterations in central components related to neural drive and motor unit activation, and peripheral components involving processes at or beyond the neuromuscular junction, including excitation–contraction coupling and muscle contractile properties (Enoka and Duchateau, 2016; Tornero-Aguilera et al., 2022). Research using disuse or immobilization models has yielded contrasting findings: some studies indicate that reduced muscle use induces alterations in central and peripheral components, including neural drive, motor unit activation, and neuromuscular junction function (Hainaut and Duchateau, 1989; Inns et al., 2022; Sergeeva et al., 2024; Piasecki, 2025), whereas others suggest that chronic disuse may preserve or even enhance neuromuscular propagation, potentially mitigating fatigue-related impairments (Fuglevand et al., 1995; Inns et al., 2022). These divergent observations underscore the need for further research to clarify how immobilization affects the specific mechanisms underlying performance fatigability.

As a result, the physiological mechanisms underlying immobilization-induced alterations in performance fatigability remain poorly understood. While muscle atrophy and neural adaptations are frequently implicated, their relative contributions to changes in fatigability have not been clearly established. A comprehensive evaluation of central and peripheral neuromuscular components is therefore necessary to elucidate the mechanisms responsible for immobilization-induced changes in performance fatigability.

From a sports performance perspective, this knowledge gap is particularly relevant. Team-sport athletes must repeatedly generate high levels of force while resisting fatigue during successive high-intensity efforts. However, limited evidence exists regarding how short-term immobilization affects performance fatigability and the underlying neuromuscular determinants in trained individuals, who may respond differently to immobilization compared with untrained or clinical populations.

Therefore, the purpose of this study was to investigate the effects of three weeks of arm immobilization on performance fatigability and its central and peripheral neuromuscular components in trained team-sport athletes. We hypothesized that short-term immobilization would lead to an increase in performance fatigability, primarily driven by impairments in neuromuscular function rather than muscle atrophy alone.

## Methods

### Ethical approval

All experimental procedures were conducted on human participants. Written informed consent was obtained from all participants prior to inclusion in the study. The study conformed to the standards set by the Declaration of Helsinki (2013 revision). Participants were recruited and enrolled between March 2026 and April 2026 (the first participant was enrolled in March 2026); all participants had therefore completed the study before registration. The trial was retrospectively registered with the Pan African Clinical Trials Registry on 16 July 2026 (PACTR202607531230552).

### Participants

Thirty competitive team-sport athletes were assessed for eligibility. Six athletes were excluded before randomization: four did not meet the eligibility criteria and two declined to participate. Consequently, 24 athletes were enrolled and randomized, including 8 handball players, 9 basketball players, and 7 volleyball players. The final sample comprised 11 females and 13 males. All participants were right-handed according to a standardized handedness questionnaire (Bryden, 1977). Participant progression through the study is presented in the CONSORT flow diagram (**Figure 1**), and participant baseline characteristics are presented in **Table 1**.

**Figure 1:**
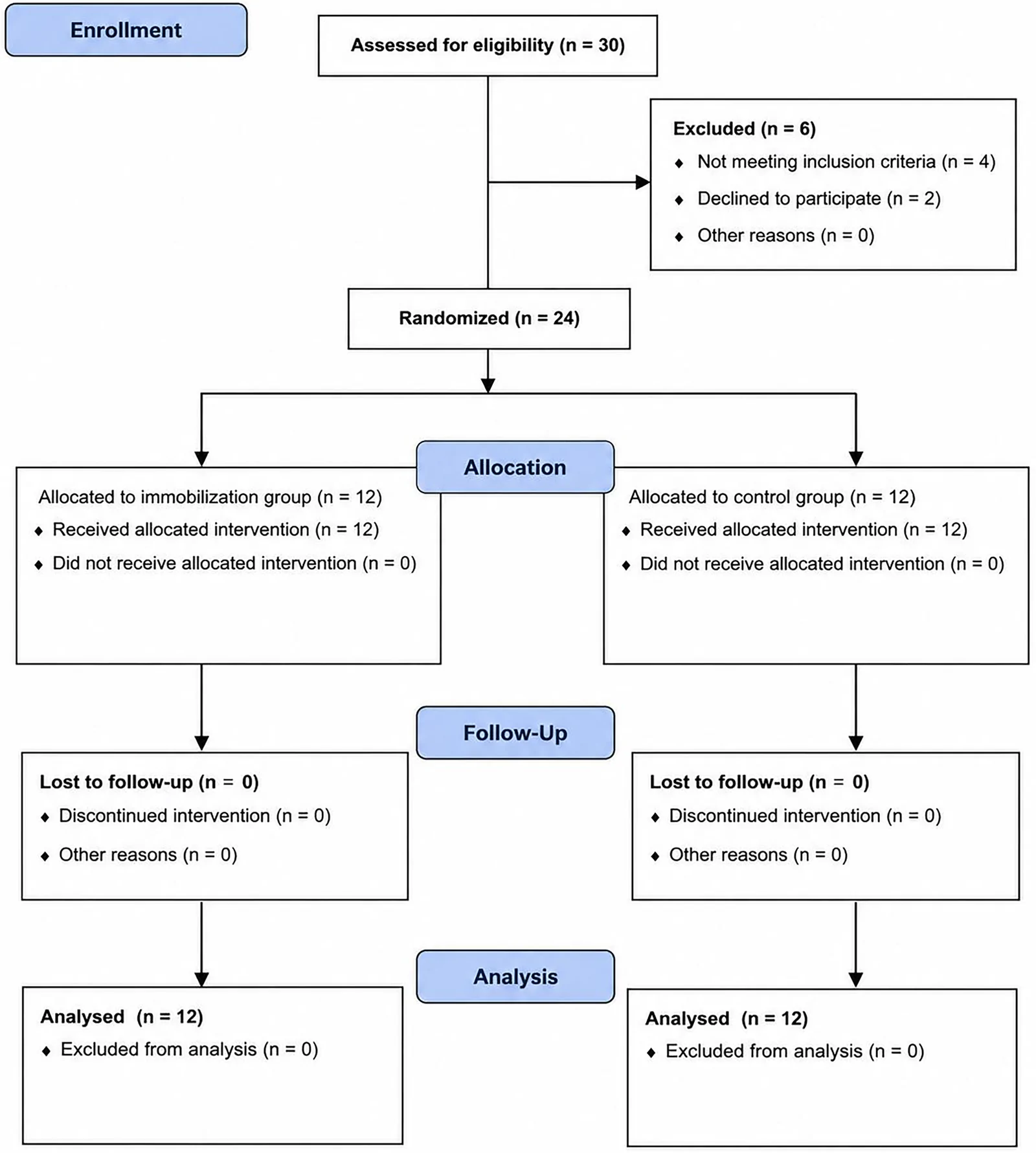
CONSORT participant-flow diagram.

**Table 1:** Descriptive characteristics of the participants.

	Immobilization group	Control group
Sex, male/female	6/6	7/5
Age (y)	21.3 ± 2.1	22.1 ± 1.9
Height (cm)	171.2 ± 6.3	172.5 ± 5.9
Weight (kg)	70.5 ± 5.8	68.9 ± 6.2
BMI (kg/m²)	24.10 ± 1.5	23.28 ± 1.4
Handball players, n	4	4
Basketball players, n	4	5
Volleyball players, n	4	3
Training experience, years	9.2 ± 3.5	8.5 ± 2.1
Weekly training volume, h/week	6.9 ± 1.5	6.5 ± 0.8

Values are mean ± SD or counts (n). BMI, body mass index. Training experience and weekly training volume refer to habitual training before the study.

Participants were eligible if they were aged 18 years or older; were competitive male or female athletes participating in handball, basketball, or volleyball; were right-handed; were able to provide written informed consent; and were able to complete maximal voluntary contraction and sustained isometric elbow-flexor exercise testing. Participants were excluded if they had a current upper-limb musculoskeletal injury or pain; a history of neurological, neuromuscular, cardiovascular, or metabolic disease; previous surgery or major injury affecting the tested upper limb; contraindications to percutaneous electrical nerve stimulation; use of medication or treatment likely to affect neuromuscular function or fatigue responses; inability to comply with the immobilization protocol or study testing procedures; or participation in structured physical training during the intervention period.

Participants were randomly assigned to either the immobilized group (IMMO) (n = 12) or the control group (CG) (n = 12). Sex distribution (6 males/6 females in IMMO vs 7 males/5 females in CG; Fisher’s exact test, p = 1.00), sport distribution, training experience and weekly training volume are reported for each group in **Table 1**. During the 3-week intervention period, participants in the IMMO group had their non-dominant arm (left) immobilized in a standardized arm sling (Sutherland Medical, Melbourne, Australia) for 12 to 14 hours each day during their waking hours, following a previously established slinging protocol (Parcell et al., 2000; Miles et al., 2005). Control group participants did not receive any intervention. The study was conducted during the off-season, and all athletes were instructed to refrain from training and structured physical activity throughout the experimental period to ensure that training did not interfere with the effects of immobilization.

### Experimental design

This study was a two-arm, parallel-group randomized controlled trial. The study is reported in accordance with the CONSORT guidelines. Participants reported to the laboratory on three occasions: one familiarization session and two experimental sessions, during which they performed the fatiguing exercise protocol (**Figure 2**).

**Figure 2:**
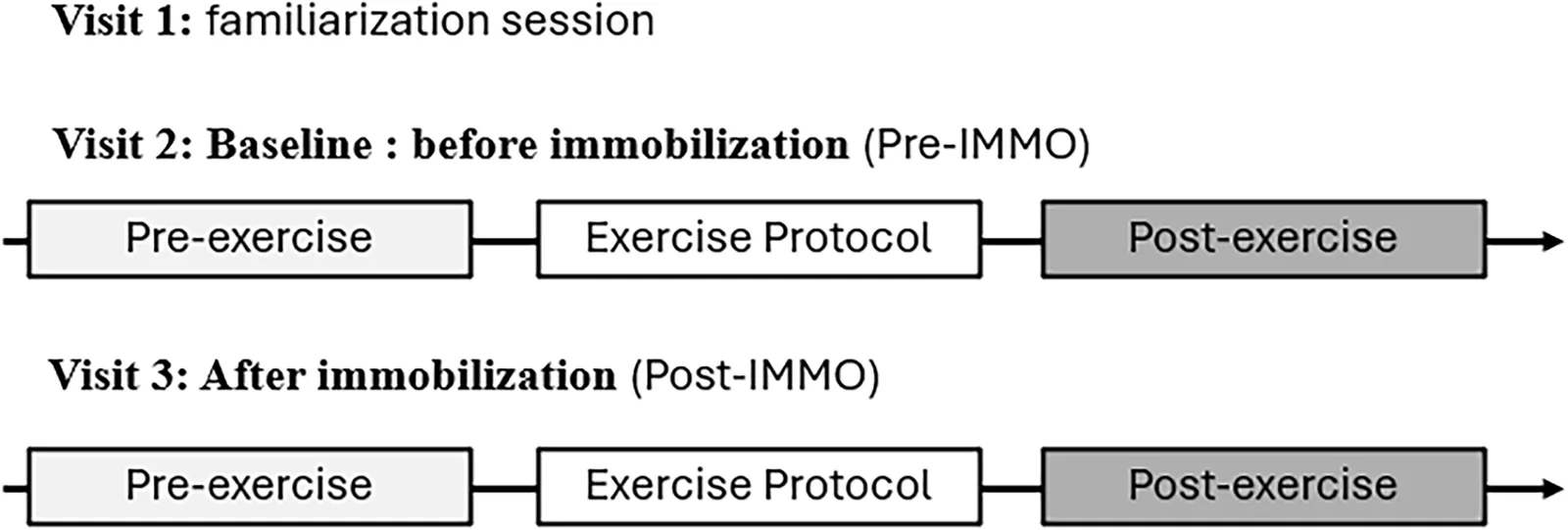
Experimental design and assessment timeline.

### Admission visit

Before the experimental session, participants attended a familiarization session during which they were introduced to the experimental procedures. On arrival at the laboratory, anthropometric variables were measured, and each subject learned to achieve and maintain maximal effort using an isometric dynamometer. The participants were also assessed for physical activity levels, and arm dominance. Participants were allocated in a 1:1 ratio to either the control group (CG) or immobilized group (IMMO) using block randomization generated via an online random number generator (random.org).

Randomization was performed after the familiarization session, so that group allocation could not influence enrollment or familiarization. Because the sling was visible, neither participants nor the investigators who conducted testing could be blinded to group allocation. To limit detection bias, all force, EMG and stimulation variables were obtained with the same standardized procedures and analysis settings for both groups and both experimental sessions.

### Interventional visit

Before and after the 3-week intervention period, the IMMO and CG participants participated in the experimental session. Both experimental sessions were conducted in the same laboratory and at the same time of day, with the same equipment, participant positioning and testing sequence. All participants refrained from training and structured physical activity throughout the experimental period (see Participants); however, sleep, nutrition, hydration, and caffeine intake were not formally standardized or recorded before testing. Participants performed a standardized warm-up consisting of several submaximal elbow flexor muscles contractions. Participants were positioned on an isometric dynamometer equipped with a cuff attached to a strain gauge. This cuff was adjusted at the level of the wrist using a non-compliant Velcro strap for continuous recording of isometric force. The non-dominant arm was placed at 90° of elbow flexion, with the elbow resting on a padded support to ensure comfort and stability. The wrist was maintained in a neutral position between supination and pronation. The hand, wrist, and forearm were secured in an appropriately sized orthosis. To minimize compensatory movements, the chest, thighs and hips were firmly stabilized using non-elastic safety straps, preventing lateral, vertical, or forward displacement during testing. Then, they were requested to perform a constant-force sustained isometric contraction at 50% of their maximal voluntary isometric contraction (MVC) until exhaustion. This sustained contraction was performed on an isometric dynamometer (Good Strength, Metitur, Finland) with the non-dominant arm positioned so that the elbow joint was maintained at 90° (0°=full extension). During the exercise, each subject was able to visualize force feedback on a computer screen. To support the subjects and maintain the force at the target level as long as possible, visual guidelines placed at ±2 N around the target force level, were set on the computer screen, and the area defined by the two guidelines was shaded with visible color. The subjects were then asked to keep the curve representing the force production within the shaded area and then the endurance time, defined as the “time limit” (Tlim), was recorded in seconds. Participants were verbally encouraged to maintain the target force level for as long as possible. Exhaustion was reached when the participant voluntarily stopped the exercise or failed to maintain force at the target level for more than 5 s. The target force was recalculated at each experimental visit as 50% of the MVC measured during that visit. Consequently, changes in MVC across visits resulted in corresponding changes in the absolute force required during the sustained contraction.

### Assessment of neuromuscular function

The maximal voluntary contraction (MVC) force was measured using an isometric dynamometer. Participants were seated with their elbow flexed at 90° and with their hands in a supinated position. The dynamometer was positioned on the participant’s forearm at the level of the wrist. The participant was then instructed to flex the elbow against the dynamometer as forcefully as possible for 3 s. Three attempts, with a 2 min rest between each attempt were performed. The value recorded was the highest value obtained after the three trials.

A constant current stimulator (Digitimer Limited, Hertfordshire, UK) delivered single square-wave stimuli of 1 ms duration with maximal voltage of 400 V percutaneously over the musculocutaneous nerve. The most proximal primary motor branch of the biceps brachii, located at a mean distance of 122 ± 12 mm distal to the coracoid process on the medial aspect of the upper arm, was targeted for stimulation (Yang et al., 1995). Subsequently, stimulation was delivered via surface electrodes with a 10-mm cathode (self-adhesive electrode: Ag-AgCl) placed directly over this primary motor branch, and a 35-mm anode (self-adhesive stimulation electrode (Compex Medical SA, Ecublens, Switzerland)) located over the medial aspect of the distal biceps tendon, just proximal to the elbow crease. Optimal stimulation intensity was determined from M-wave and force measurements before each testing session. Stimulation of the musculocutaneous nerve was carefully optimized to ensure selective activation of the elbow flexor muscles. After electrode placement, the stimulation site and intensity were systematically adjusted to elicit a clear and isolated elbow flexion twitch. Specific attention was given to the absence of any visible triceps brachii contraction or elbow extension torque during stimulation. The stimulation intensity was increased by 5 mA until there was no further increase in peak twitch force (i.e., plateau in elbow flexors twitch force). During the subsequent testing procedures, the intensity was set to 150% of this intensity (supramaximal intensity) to overcome the potential confounding effect of axonal hyperpolarization (Burke, 2002). The absolute stimulation intensity was 49.00 ± 16.60 mA (CG) and 47.90 ± 17.40 mA (IMMO) before immobilization, and 48.50 ± 16.80 mA (CG) and 48.80 ± 17.00 mA (IMMO) after immobilization, with no significant differences between groups or time points (p > 0.05). This indicates that stimulation intensity was consistent across all measurements. For each MVC, two electrical nerve stimulations were delivered. The first stimulation, delivered during the MVC, was named the superimposed twitch, and the second stimulation, delivered 3 s after the MVC, was named the potentiated twitch (Q_tw,pot_). This delay provided the opportunity to obtain a potentiated mechanical response which reduces the variability of the measurement compared to a non-potentiated twitch (Kufel et al., 2002). Performance fatigability (peripheral + central) was determined as the difference in MVC from baseline to post exercise (ΔMVC%). Peripheral components of the biceps brachii were quantified as the difference in Q_tw,pot_ from baseline to post exercise (ΔQ_tw,pot_%). For central components, voluntary activation (VA) was calculated using both superimposed and potentiated twitch amplitudes as follow (Merton, 1954):


$$\text{Voluntary activation }\left(\%\right)\;=\;\left[1\;-\;\left(\text{Superimposed twitch}/{\text{Q}}_{\text{tw},\text{pot}}\right)\right]\;*100$$

### Electromyography

The EMG signals were recorded using bipolar silver chloride surface electrodes (Dormo Electrodes, SX-50 ECG, Spain). The recording electrodes were taped lengthwise on the skin over the biceps brachii muscle belly following SENIAM recommendations (Hermens et al., 1999), with an inter-electrode distance of 20 mm. The reference electrode was attached to the lateral epicondyle. Low impedance (Z < 5 kΩ) at the skin–electrode surface was obtained by shaving, abrading the skin with emery paper and cleaning it with alcohol. To guarantee the electrodes were placed exactly the same on subsequent visits, the position was marked with permanent ink. Electromyographic signals were amplified (Octal Bio Amp ML 138, ADInstruments, Australia) with a bandwidth frequency ranging from 10 Hz to 1 kHz (common mode rejection ratio >96 dB, gain = 1,000) and simultaneously digitized together with force signals using an acquisition card (Powerlab 16SP, ADInstruments, Australia) and the Labchart 7.0 software (ADInstruments, Australia). The sampling frequency was 2 kHz. For each Q_tw,pot_, peak-to-peak amplitude of the maximal muscle action potential (i.e. M-wave, Mmax) was measured. During the MVC trials, EMG activation of brachii muscle short head (SH) and long head (LH) were calculated using the root mean square (RMS), and the mean of the two RMS values was used. Specifically, the RMS was determined over a fixed 0.5-s window before peak force. To account for alterations in membrane excitability following exercise, raw RMS data were normalized to the maximal M-wave amplitude measured during the pre-exercise assessment of each visit.

### Immobilization

The non-dominant arm (left) was unloaded for three weeks using a sling and bandage. Previous slinging protocols used by Parcell et al. (2000) and Miles et al. (2005) effectively reduced cross-sectional area and strength after 3 and 4 weeks, respectively (Parcell et al., 2000; Miles et al., 2005). The sling held the elbow flexed (elbow at 90°) while the swathe supported the arm against the torso. The sling unloads the elbow flexor and extensor muscles, while the swathe prevents the shoulder muscles from supporting the arm and restricts shoulder mobility. Participants were asked to wear the sling and swathe during waking hours to keep their arms immobilized and to take them off only when sleeping or showering. Participants in both the IMMO and CG were also not allowed to undertake any form of physical work or drive a motor vehicle. Participants in the IMMO group were required to record the duration of sling usage (≥12–14 h/day). Any instances of sling removal were to be recorded, including the reason for removal, the length of time it was removed, as well as the specific day and time of the occurrence. These self-reported records indicated similar activity levels across participants; physical activity was not objectively monitored. The average duration of sling usage was 12.8 ± 0.7 h/day, and no differences in self-reported activity levels were observed between participants, indicating good self-reported compliance with the protocol.

### Anthropometric estimations of muscle and bone area

The estimation of muscle and bone areas (MBA) in the upper arm was derived using various anthropometric formulas. These formulas operate under the assumption that the arm, muscle, and bone compartments have a circular geometry (Gurney and Jelliffe, 1973; Heymsfield et al., 1982; Forbes, 1986). Specifically, de Koning et al. (1986) and Forbes (1986) calculated mid-arm muscle and bone areas using arm circumference measurements in combination with triceps and biceps skinfold thickness (De Koning et al., 1986; Forbes, 1986). This muscle and bone area anthropometric function (MBA.ANf) is calculated as shown below. MBA is therefore an indirect, composite estimate of the muscle-and-bone cross-sectional area of the upper arm; it does not measure muscle morphology directly, and no imaging technique (ultrasonography or magnetic resonance imaging) was used in the present study.


$$\text{MBA}.\text{ANf}=\;1/(4\pi )\;{\left[\text{extended upper−arm circumference }-(\pi /2)\left(\text{biceps skinfold}+\text{ triceps skinfold}\right)\right]}^{2}$$

### Statistical analyses

All statistical analyses were performed using R statistical software (version 4.5.1) within the RStudio environment. No formal *a priori* sample-size calculation was performed. The sample size was determined by the number of eligible competitive athletes available during the recruitment period. The relatively small sample should therefore be considered when interpreting the precision and generalizability of the findings. To indicate the effects that the design could reliably detect, a sensitivity analysis was performed. Because every interaction involving group and two-level within-subject factors is equivalent to a between-group comparison of individual contrast scores, with 12 participants per group and α = 0.05 the study had 80% power to detect interaction effects of η_p_² ≥ 0.26 (Cohen’s f ≥ 0.60; d ≥ 1.20 for the contrast scores). Smaller interaction effects may therefore have gone undetected. Parametric analyses were conducted using the afex package for mixed-model ANOVA (Singmann et al., 2016). Descriptive statistics are presented as mean ± standard deviation (SD). Prior to analysis, the normality of each dependent variable was assessed using the Shapiro–Wilk test, and homogeneity of variance between groups was verified using the Levene test. MBA and time to task failure (Tlim) were analyzed using a two-way mixed-model analysis of variance (ANOVA) with group (control vs. immobilization) as the between-subject factor and condition (before vs. after immobilization) as the within-subject factor. Neuromuscular parameters, including maximal voluntary contraction (MVC), potentiated twitch force (Q_tw,pot_), voluntary activation (VA), RMS/Mmax, and short-head (SH) and long-head (LH) M-wave amplitudes, were analyzed using a three-way mixed-model ANOVA with the factors exercise (pre- vs. post-exercise), condition (before vs. after immobilization), and group (control vs. immobilization). Because each within-subject factor had two levels, the sphericity assumption did not apply. When significant interaction effects were detected, pairwise comparisons of estimated marginal means were performed using the emmeans package (R), with Bonferroni-adjusted p-values. Pre- to post-exercise percentage changes (Δ) were calculated for each participant to illustrate the magnitude of exercise-induced fatigue before and after immobilization, but these values were not used as primary inputs for inferential analyses. Effect sizes were reported as partial eta-squared (η2) for ANOVA and interpreted as small (0.01), moderate (0.06), or large (0.14) (Cohen, 2013). Exact 95% confidence intervals (CI) for η_p_² were derived from the F statistic and its degrees of freedom using the noncentral F distribution (Steiger, 2004). For key pairwise comparisons, mean differences are reported with 95% CIs derived from the fitted mixed-model ANOVA (pooled error term). Standardized mean differences (Cohen’s d) were calculated for pairwise comparisons and interpreted using Hopkins’ thresholds: trivial (d ≤ 0.20), small (0.20 < d ≤ 0.60), moderate (0.60 < d ≤ 1.20), large (1.20 < d ≤ 2.0), very large (2.0 < d ≤ 4.0), and extremely large (d > 4.0) (Hopkins et al., 2009). Statistical significance was set *a priori* at p < 0.05. Non-significant interactions were interpreted as the absence of evidence for a differential effect, not as evidence that no effect occurred.

## Results

### Anthropometric estimations

A significant (group × immobilization) interaction effect (F _[1, 22]_ =29.014, p=0.001, ηp² = 0.805) and main effect of immobilization (F _[1, 22]_ =36.460, p<0.001, ηp² = 0.838) were found for changes in MBA values. At baseline, there were no significant differences for the MBA between the groups for the non-dominant arm (p = 0.835). After 3 weeks of immobilization, MBA values decreased significantly in the IMMO group (12.54 ± 0.4 vs 11.63 ± 0.37, p = 0.001). However, no significant changes were observed in MBA in CG following the immobilization period (12.28 ± 2.39 vs 12.23 ± 2.25, p = 0.639) (**Figure 3**).

**Figure 3:**
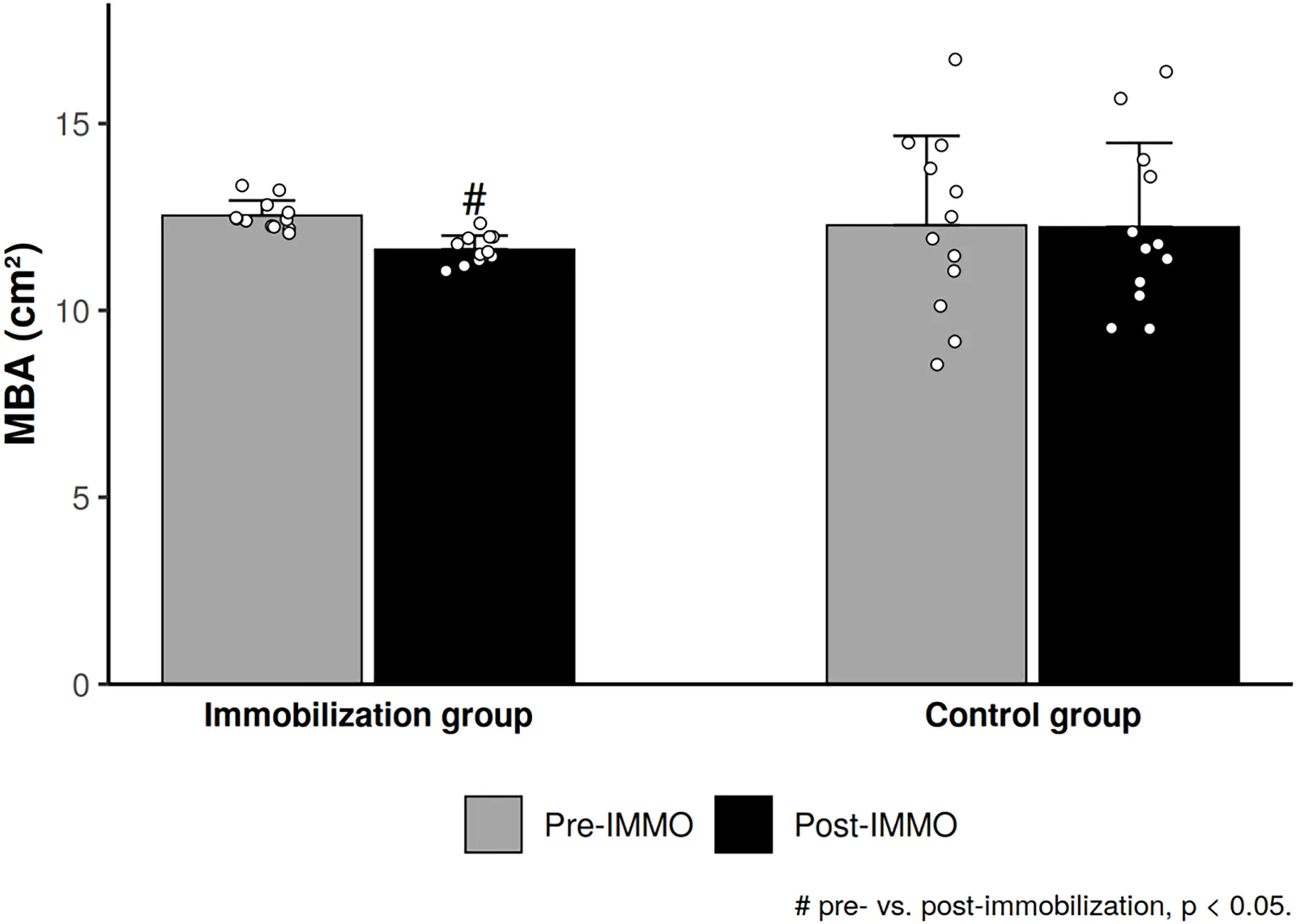
Anthropometrically estimated upper-arm muscle-and-bone area (MBA) in the control and immobilization groups before and after the immobilization period. MBA was estimated anthropometrically from arm circumference and biceps and triceps skinfolds. Bars represent group means, error bars represent SD, and open circles represent individual participants (n = 12 per group). ^#^p < 0.05 pre- vs post-immobilization within the group (Bonferroni-adjusted).

### Exercise performance

Statistical analysis revealed a significant (group × immobilization) interaction for Tlim (F[_1, 22_] = 19.965, p = 0.001, ηp² = 0.666), as well as a significant main effect of immobilization (F[1, _22_] = 26.574, p < 0.001, ηp² = 0.726). *Post-hoc* analyses showed a significant increase in Tlim following the immobilization period in the IMMO group (220.88 ± 102.7 vs 281.65 ± 99.65 s, p < 0.001), whereas no significant change was observed in baseline Tlim values in the CG (207.15 ± 86.65 vs 208.15 ± 78.35 s, p = 0.811) (**Figure 4**). Because the target force was set at 50% of the pre-exercise MVC measured at each visit, the absolute target force in the IMMO group was lower after than before immobilization (69.3 ± 11.9 vs 89.9 ± 17.5 N), whereas it was similar across conditions in the CG (89.3 ± 20.2 vs 91.6 ± 23.3 N). These values were derived from the pre-exercise MVC values in **Table 2**; thus, after immobilization the IMMO group sustained a lower absolute force for a longer time.

**Figure 4:**
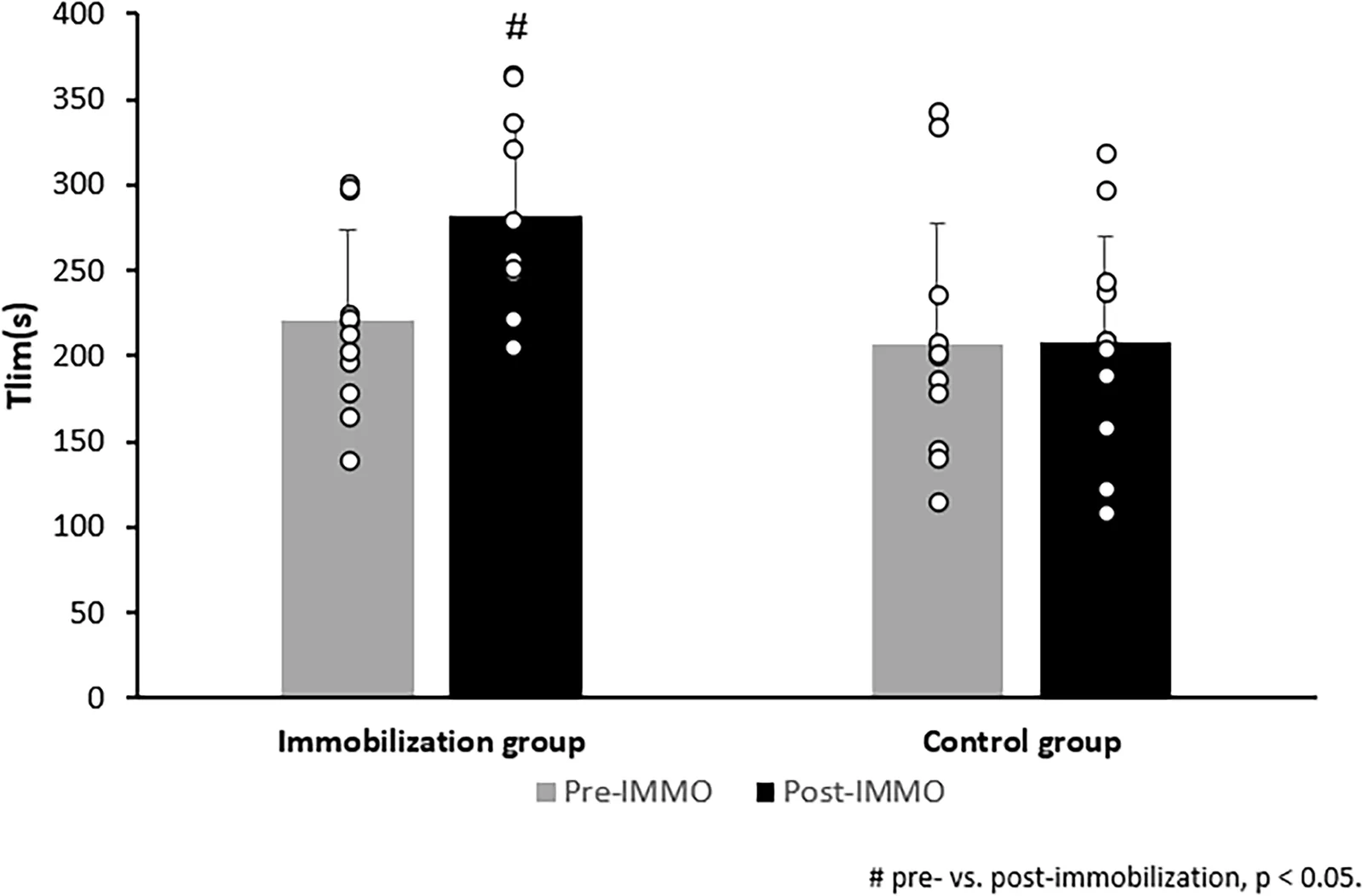
Endurance time (Tlim) in the control and immobilization groups before and after the immobilization period. Tlim is the duration of the sustained isometric elbow-flexor contraction at 50% of the MVC measured at each visit; the absolute target force was therefore lower after immobilization in the IMMO group. Bars represent group means, error bars represent SD, and open circles represent individual participants (n = 12 per group). ^#^p < 0.05 pre- vs post-immobilization within the group (Bonferroni-adjusted).

**Table 2:** Neuromuscular parameters assessed before and after exercise, and before and after the immobilization period (mean ± SD).

Units	Immobilisation				Control			
	Pre immobilization		Post immobilization		Pre immobilization		Post immobilization	
	Pre ex	Post ex	Pre ex	Post ex	Pre ex	Post ex	Pre ex	Post ex
MVC (N)	179.76 ± 35.07	122.41 ± 27.32^*^	138.56 ± 23.87^#α^	118.74 ± 23.05^*^	178.62 ± 40.32	132.61 ± 35.19^*^	183.27 ± 46.62	136.07 ± 38.34^*^
Δ%		-31.46 ± 10.22		-14.36 ± 7.75^#^		-25.75 ± 9.07		-25.50 ± 8.73
Q_tw,pot_ (N)	34.32 ± 14.42	18.24 ± 5.48^*^	30.39 ± 6.64	25.67 ± 5.60	33.36 ± 7.58	20.81 ± 10.74^*^	33.73 ± 7.84	21.05 ± 5.13^*^
Δ%		-38.91 ± 20.09		-15.15 ± 8.73		-36.12 ± 19.54		-34.91 ± 19.66
VA (%)	86.94 ± 5.10	81.99 ± 4.52^*^	81.03 ± 4.37	79.37 ± 4.44	87.71 ± 2.52	82.09 ± 6.22^*^	87.77 ± 3.32	82.68 ± 4.35^*^
Δ%		-5.51 ± 1.87		-1.98 ± 1.34		-6.42 ± 1.38		-5.74 ± 1.85
M-waves LH (mV)	8.05 ± 1.78	6.56 ± 3.27^*^	7.83 ± 2.87	6.78 ± 3.22	7.57 ± 1.64	6.13 ± 1.76^*^	7.43 ± 1.61	5.98 ± 1.45^*^
Δ%		-15.05 ± 8.33		-8.90 ± 5.37		-19.80 ± 8.96		-18.96 ± 11.42
M-waves SH (mV)	7.19 ± 2.53	6.22 ± 2.24^*^	7.77 ± 2.19	6.37 ± 3.38^*^	7.43 ± 2.54	6.13 ± 2.64^*^	7.24 ± 1.97	6.17 ± 2.16^*^
Δ%		-11.67 ± 9.11		-21.36 ± 13.53		-16.37 ± 9.55		-16.52 ± 12.32
RMS/Mmax (a.u.)	0.10 ± 0.05	0.08 ± 0.03^*^	0.10 ± 0.09	0.08 ± 0.03	0.10 ± 0.07	0.08 ± 0.05^*^	0.11 ± 0.05	0.08 ± 0.04^*^
Δ%		-15.56 ± 7.24		-9.04 ± 5.25		-16.09 ± 7.93		-16.15 ± 8.10

MVC, maximal voluntary contraction; Q_tw,pot_, biceps brachii potentiated twitch force; VA, voluntary activation of biceps brachii motor units; SH, short head; LH, long head; *p < 0.05 versus pre-exercise within the same group and experimental condition; ^#^p < 0.05 versus the corresponding pre-immobilization value; α: p < 0.05 versus the corresponding control-group value. Δ%, individual pre- to post-exercise percentage change (mean ± SD; descriptive). RMS/M_max_, root-mean-square EMG amplitude normalized to the maximal M-wave (arbitrary units, a.u.); M-waves, peak-to-peak amplitude of the maximal compound muscle action potential. Pre ex/Post ex, before/after the sustained contraction at 50% MVC; Pre/Post immobilization, before/after the 3-week intervention period.

Statistical analysis of maximal voluntary contraction (MVC) revealed a significant group × condition × exercise interaction (F(1,22) = 15.74, p < 0.001, η_p_² = 0.417, 95% CI 0.10–0.62), indicating that the magnitude of exercise-induced MVC changes differed according to both group and immobilization condition. A significant main effect of exercise was also observed (F (1,22) = 194.87, p < 0.001, ηp² = 0.899), confirming a marked reduction in MVC following the fatiguing exercise protocol across conditions. In addition, significant group × condition (F (1,22) = 6.37, p = 0.019, ηp² = 0.225) and condition × exercise interactions (F (1,22) = 13.86, p = 0.001, ηp² = 0.386) were detected. *Post hoc* comparisons revealed that pre-exercise MVC values decreased significantly following the immobilization period in the IMMO group (179.76 ± 35.07 vs 138.56 ± 23.87, p < 0.001; mean difference −41.2 N, 95% CI −59.4 to −23.0), and after immobilization pre-exercise MVC was lower in the IMMO group than in the CG (mean difference −44.7 N, 95% CI −75.8 to −13.7). Following exercise MVC decreased significantly from pre- to post-exercise in both groups and conditions (p < 0.001). However, the magnitude of the exercise-induced MVC decline differed according to group and immobilization condition. In the IMMO group, the exercise-induced reduction in MVC was smaller after than before the immobilization period (−14.4 ± 7.8% vs −31.5 ± 10.2%; pre- vs post-exercise: 138.56 ± 23.87 vs 118.74 ± 23.05 N, p = 0.001, mean difference −19.8 N, 95% CI −30.0 to −9.6; and 179.76 ± 35.07 vs 122.41 ± 27.32 N, p < 0.001, mean difference −57.4 N, 95% CI −70.2 to −44.5, respectively), noting that the post-immobilization task was performed at a lower absolute force. In contrast, no significant difference in the magnitude of MVC decline between the pre- and post-immobilization conditions was observed in the CG (178.62 ± 40.32 vs 132.61 ± 35.19 N, p < 0.001, mean difference −46.0 N, 95% CI −58.9 to −33.1; and 183.27 ± 46.62 vs 136.07 ± 38.34 N, p < 0.001, mean difference −47.2 N, 95% CI −57.4 to −37.0, respectively) (**Figure 5**, **Table 2**).

**Figure 5:**
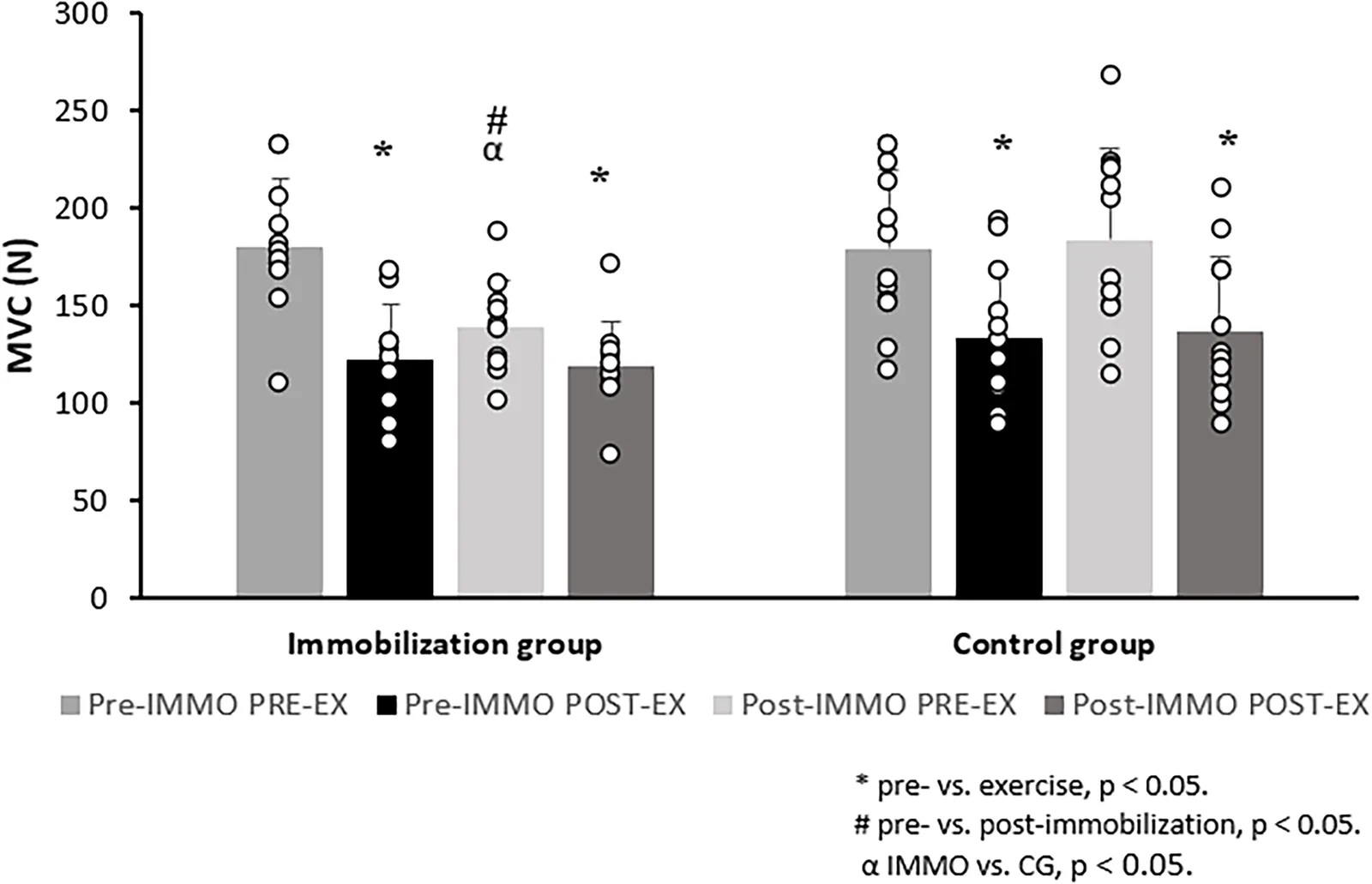
Maximal voluntary contraction (MVC) values before and after exercise, during the pre- and post-immobilization assessments in the control and immobilization groups. Bars represent group means, error bars represent SD, and open circles represent individual participants (n = 12 per group). *p < 0.05 pre- vs post-exercise; ^#^p < 0.05 vs the corresponding pre-immobilization value; α, p < 0.05 vs the control group (Bonferroni-adjusted).

### Central and peripheral fatigue

#### Voluntary activation

The three-way ANOVA revealed a significant main effect of exercise on voluntary activation (VA) (F (_1,22_) = 46.88, p < 0.001, ηp² = 0.681). No significant group × condition × exercise interaction was observed (F(1,22) = 2.48, p = 0.130, η_p_² = 0.10, 95% CI 0.00–0.35). Following exercise, VA decreased significantly in the CG before and after the immobilization period (87.71 ± 2.52% vs. 82.09 ± 6.22%, p < 0.001, d = 1.18; and 87.77 ± 3.32% vs. 82.68 ± 4.35%, p = 0.001, d = 1.32, respectively), as well as in the IMMO group before immobilization (86.94 ± 5.10% vs. 81.99 ± 4.52%, p = 0.001, d = 1.03). In the IMMO group after immobilization, the exercise-induced change was not statistically significant (81.03 ± 4.37% vs. 79.37 ± 4.44%, p = 0.14, d = 0.38). Given the absence of a significant three-way interaction, these within-condition differences do not indicate a differential effect of immobilization on the VA response to exercise (**Figure 6**, **Table 2**).

**Figure 6:**
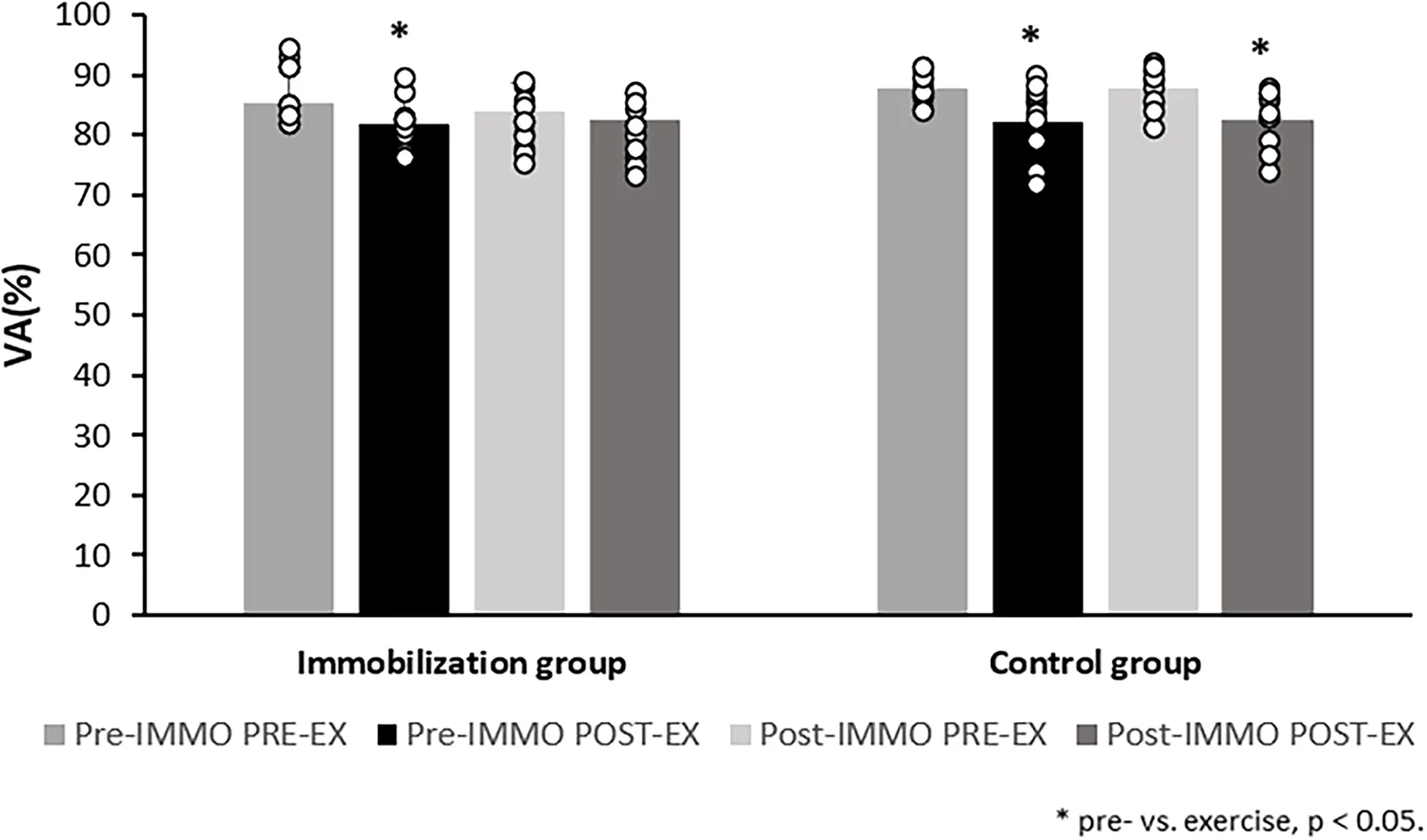
Voluntary activation (VA) values before and after exercise, during the pre- and post-immobilization assessments in the control and immobilization groups. Bars represent group means, error bars represent SD, and open circles represent individual participants (n = 12 per group). *p < 0.05 pre- vs post-exercise (Bonferroni-adjusted). The group × condition × exercise interaction was not significant (p = 0.130).

### Potentiated twitch (Q_tw,pot_)

A significant main effect of exercise (p < 0.001) and a significant group × condition × exercise interaction (F(1,22) = 4.58, p = 0.044, η_p_² = 0.17, 95% CI 0.00–0.42) were observed for Qtw,pot.

Qtw,pot decreased significantly following exercise in both groups before immobilization and in the control group after immobilization (control: 33.36 ± 7.58 vs 20.81 ± 10.74, p < 0.001, d = 1.35; and 33.73 ± 7.84 vs 21.05 ± 5.13, p < 0.001, d = 1.91; IMMO before immobilization: 34.32 ± 14.42 vs 18.24 ± 5.48, p < 0.001, d = 1.47), whereas the post-exercise decrease was smaller and not statistically significant in the IMMO group after immobilization (30.39 ± 6.64 vs 25.67 ± 5.60, p = 0.15, d = 0.77). The significant interaction indicates that the exercise-induced decline in Q_tw,pot_ differed according to group and condition; however, the lower bound of the 95% CI for η_p_² approached zero, so the magnitude of this effect is imprecisely estimated (**Figure 7**, **Table 2**).

**Figure 7:**
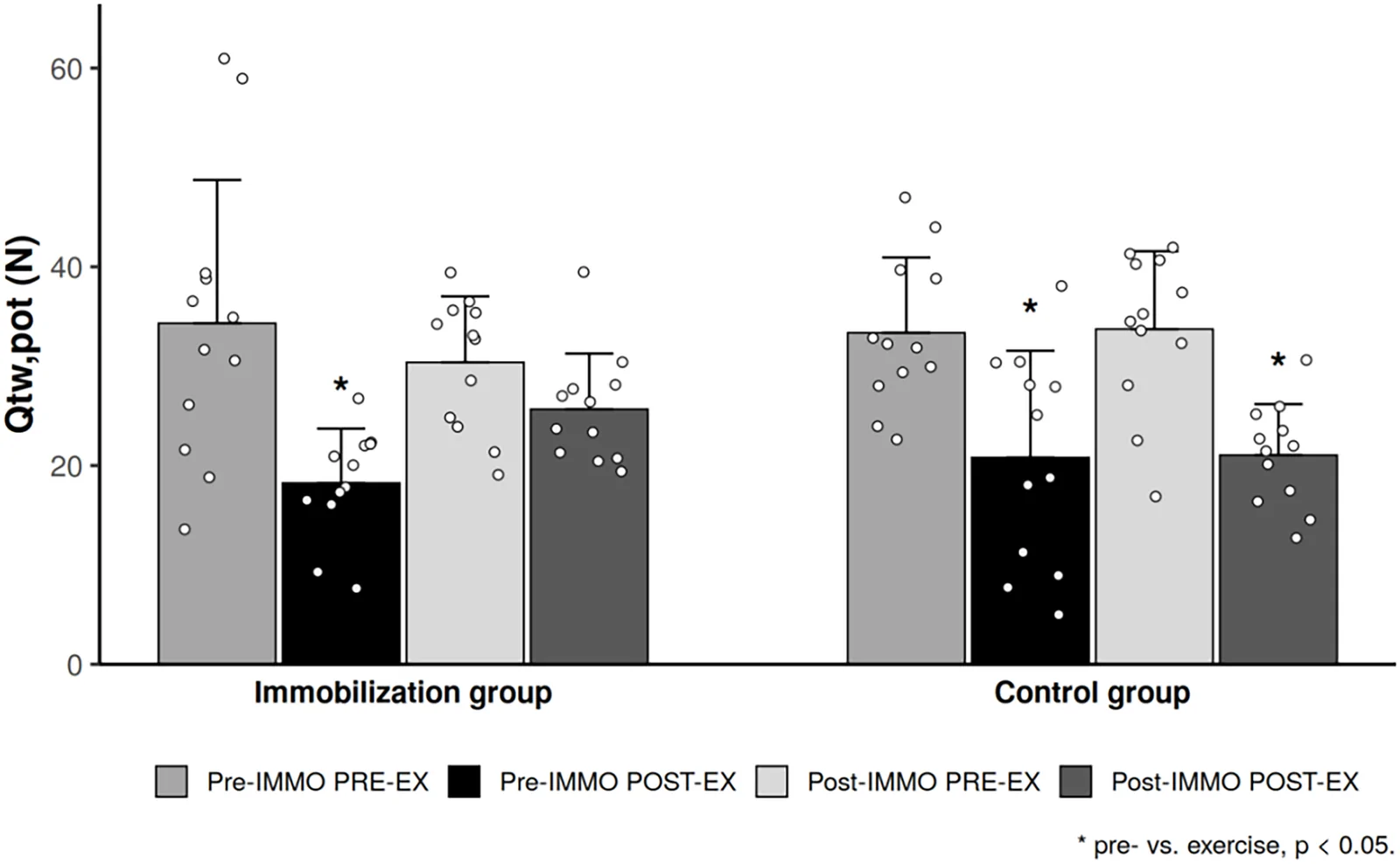
Potentiated twitch force (Q_tw,pot_) values before and after exercise, during the pre- and post-immobilization assessments in the control and immobilization groups. Bars represent group means, error bars represent SD, and open circles represent individual participants (n = 12 per group). *p < 0.05 pre- vs post-exercise (Bonferroni-adjusted).

### RMS/Mmax

The three-way ANOVA revealed a significant main effect of exercise on RMS/Mmax (F(_1,22_) = 11.664, p = 0.002, ηp² = 0.346), indicating an overall reduction following the fatiguing task. No significant group × condition × exercise interaction was observed (F(1,22) = 0.77, p = 0.391, η_p_² = 0.03, 95% CI 0.00–0.25). RMS/Mmax decreased significantly following exercise in the CG before and after immobilization (0.10 ± 0.07 vs. 0.08 ± 0.05, p < 0.001; and 0.11 ± 0.05 vs. 0.08 ± 0.04, p < 0.001, respectively) and in the IMMO group before immobilization (0.10 ± 0.05 vs. 0.08 ± 0.03, p < 0.001). In the IMMO group after immobilization, the pre- to post-exercise difference was not statistically significant (0.10 ± 0.09 vs. 0.08 ± 0.03, p = 0.15). Overall, no differential effect of immobilization on the RMS/M_max_ response to exercise was detected (**Figure 8**, **Table 2**).

**Figure 8:**
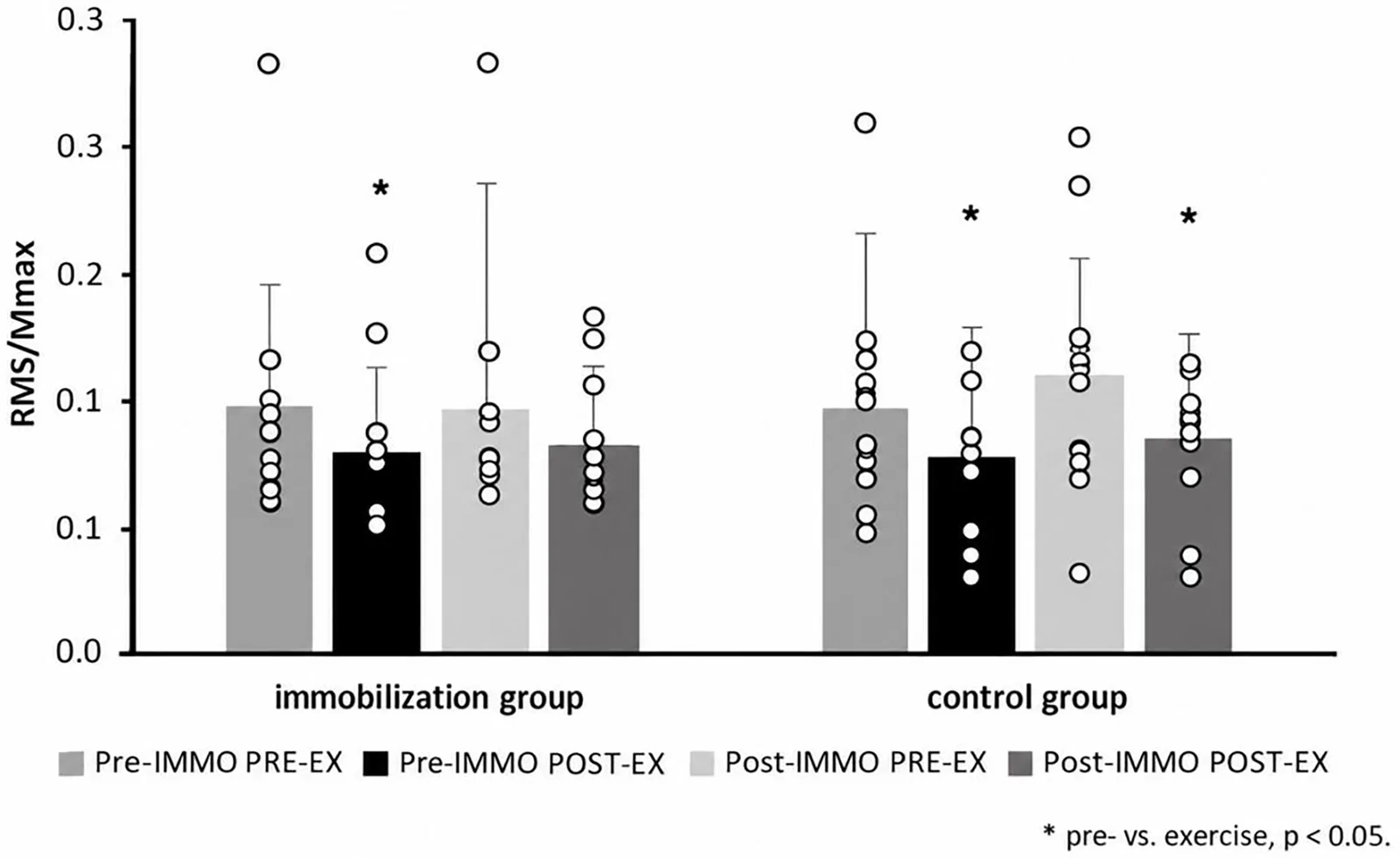
RMS/Mmax values before and after exercise, during the pre- and post-immobilization assessments in the control and immobilization groups. RMS/Mmax, root-mean-square EMG amplitude of the biceps brachii normalized to the maximal M-wave (arbitrary units). Bars represent group means, error bars represent SD, and open circles represent individual participants (n = 12 per group). *p < 0.05 pre- vs post-exercise (Bonferroni-adjusted). The group × condition × exercise interaction was not significant (p = 0.391).

### M-waves

Statistical analysis revealed a significant main effect of exercise on M-wave amplitude for both the SH (F(1,22) = 23.45, p < 0.001, ηp² = 0.515) and (LH) (F(1,22) = 11.55, p = 0.002, ηp² = 0.344), indicating a reduction in M-wave amplitude following the exercise protocol across groups and conditions. No significant group × condition × exercise interaction was observed (F (1,22) = 4.89, p>0.05).

Following exercise, SH M-wave amplitude decreased significantly in the CG before and after immobilization (7.43 ± 2.54 vs 6.13 ± 2.64, p = 0.04, d = 0.50; and 7.24 ± 1.97 vs 6.17 ± 2.16, p = 0.02, d = 0.52, respectively), as well as in the IMMO group before and after immobilization (7.19 ± 2.53 vs 6.22 ± 2.24, p = 0.04, d = 0.41; and 7.77 ± 2.19 vs 6.37 ± 3.38, p = 0.004, d = 0.49, respectively).

Similarly, LH M-wave amplitude decreased significantly in the CG before and after immobilization (7.57 ± 1.64 vs 6.13 ± 1.76, p = 0.04, d = 0.85; and 7.43 ± 1.61 vs 5.98 ± 1.45, p = 0.04, d = 0.95, respectively), as well as in the IMMO group before immobilization (8.05 ± 1.78 vs 6.56 ± 3.27, p = 0.04, d = 0.57). In contrast, no significant decrease in LH M-wave amplitude following exercise was observed in the IMMO group after immobilization (7.83 ± 2.87 vs 6.78 ± 3.22, p = 0.93, d = 0.34). (**Table 2**).

## Discussion

The primary aim of this study was to examine the effects of short-term upper-limb immobilization on neuromuscular function and exercise-induced fatigue in team-sport athletes. The present findings demonstrate that three weeks of arm immobilization induced significant neuromuscular alterations characterized by reductions in anthropometrically estimated muscle-and-bone area and maximal strength together with changes in the fatigue response during sustained submaximal exercise. Anthropometrically estimated MBA decreased by 7.3% in the IMMO group. Because this measure is derived from arm circumference and skinfold thickness and represents a composite estimate of muscle-and-bone area, the finding should not be interpreted as direct confirmation of muscle atrophy. This reduction was accompanied by a marked fall in baseline maximal voluntary contraction (MVC), in line with previous evidence on the detrimental effects of short-term disuse. In contrast, endurance time (Tlim) during the relative-intensity task increased following immobilization (+27.5%), although the task was performed at a lower absolute force, and the magnitude of the exercise-induced decline in MVC was attenuated after immobilization (−31.5% pre- vs −14.4% post-immobilization). The magnitude of exercise-induced reduction in potentiated twitch force (Qtw,pot) was likewise attenuated (IMMO: −38.9% pre- vs −15.2% post-immobilization), indicating a smaller peripheral fatigue response. No significant interaction was detected for voluntary activation, RMS/Mmax or M-wave amplitude. Because the fatigue task was set at a relative intensity (50% MVC), however, this peripheral effect could be explained by the lower absolute force produced after immobilization rather than by an intrinsic gain in fatigue resistance.

### Immobilization effects on neuromuscular function

Three weeks of upper-limb immobilization resulted in significant reductions in both MVC and anthropometrically estimated upper-arm MBA relative to baseline (−22.9% and −7.3%, respectively), indicating a clear loss of force-generating capacity. These findings are consistent with previous studies reporting reductions in isometric strength of the elbow flexor muscles following short-term immobilization, including decreases observed after two weeks (Vaughan, 1989; Seo et al., 2024) and four weeks (Yue et al., 1997; Valdes et al., 2021) of limb disuse. Valdes et al. (2021) showed that four weeks of arm sling immobilization in healthy young adults reduced MVC, muscle activity, and arm circumference of the immobilized limb (Valdes et al., 2021). Pearce et al. (2013) reported decreases in elbow flexor strength, muscle thickness, and corticospinal excitability after three weeks of sling immobilization (Pearce et al., 2013). Together, these results underline the sensitivity of skeletal muscle to relatively brief periods of mechanical unloading.

The decline in MVC observed here is likely attributable to both structural and neural adaptations to immobilization. Muscle atrophy following reduced mechanical loading is a well-established consequence of disuse and is associated with suppressed protein synthesis and muscle fiber atrophy, particularly in type II fibers that contribute substantially to force production (Bokov et al., 2025); such effects are often greater during upper-limb than lower-limb disuse (Campbell et al., 2019). Accordingly, the concomitant reductions in estimated MBA and MVC observed in the present study are consistent with impaired force-generating capacity and illustrate the rapid deterioration of muscle function that can occur even after short periods of unloading. However, muscle morphology and fiber-type composition were not measured directly, and pre-exercise VA was descriptively lower in the IMMO group after immobilization (81.03 ± 4.37% vs 86.94 ± 5.10%), so a neural contribution to the MVC loss cannot be excluded.

Regarding VA, the present results did not reveal significant immobilization-related interaction effects; given the sample size, however, a moderate change in VA cannot be excluded. Previous data on neural adaptation to immobilization are inconsistent. Reductions in VA have been reported after wrist immobilization (Clark et al., 2009; Clark et al., 2010; Clark et al., 2014), whereas Magnus et al. (2010) found no change after elbow flexor immobilization (Magnus et al., 2010). These discrepancies underline the complexity of the neural response and suggest that immobilization duration, the muscle group involved, and study design all shape the magnitude of any adaptation. A reduction in VA is commonly interpreted as reflecting a diminished ability of the central nervous system to fully recruit the motoneuron pool during voluntary effort. Proposed mechanisms include reduced corticospinal excitability (Taylor et al., 2016), altered Ia-mediated reciprocal inhibition, greater inhibitory feedback from group III and IV afferents [Foley and Kalmar, 2019], and intrinsic changes in motoneuron properties (Gandevia et al., 1996). Given the absence of significant interaction effects for VA, the role of these mechanisms remains speculative and cannot be confirmed from the present data.

At the peripheral level, M-wave amplitude and normalized EMG (RMS/Mmax) showed no significant interaction, providing no evidence that short-term immobilization altered sarcolemmal excitability or neuromuscular propagation, although smaller effects cannot be excluded with the present sample size. Potentiated twitch force (Qtw,pot), in contrast, did show a significant interaction; because this effect appeared specifically in response to the fatigue task rather than at rest, it is considered in the next section. This observation is compatible with Semmler et al. (2000), who reported relative preservation of biceps brachii contractile properties after short-term immobilization despite reduced maximal force (Semmler et al., 2000).

### Immobilization effects in exercise-induced neuromuscular fatigue

Neuromuscular fatigue of the elbow flexor muscles was assessed using a sustained submaximal isometric contraction performed at 50% of MVC held to task failure. After immobilization, endurance time increased significantly (220.88 ± 102.7 vs 281.65 ± 99.65 s, pre- vs post-immobilization; +27.5%), indicating that participants sustained the relative-intensity task for longer despite the concurrent loss of maximal strength. This dissociation between reduced maximal force capacity and prolonged endurance is, however, largely expected under a relative-intensity design. A key consideration when interpreting it is the relative nature of the task: because intensity was fixed at 50% MVC, the post-immobilization fall in MVC lowered the absolute force required during the task, and lower absolute force is associated with reduced intramuscular pressure, which can improve perfusion and delay the build-up of metabolic by-products during sustained contractions (Semmler et al., 2000).

The longer Tlim after immobilization may therefore stem, at least in part, from this reduced absolute demand rather than from an intrinsic change in fatigue-resistance mechanisms, consistent with evidence that endurance during sustained contractions depends strongly on absolute load and the associated circulatory constraints (Shaffer et al., 2000; Sergeeva et al., 2024). Consistent with this interpretation, the approximate contraction impulse (group-mean target force × group-mean Tlim) was similar before and after immobilization in the IMMO group (≈19.9 vs ≈19.5 kN·s) and in the CG (≈18.5 vs ≈19.1 kN·s), suggesting that the longer Tlim largely offset the lower absolute force. Because this estimate is based on group means rather than individual force–time integrals, it should be regarded as indicative only. Turning to peripheral fatigue, post-exercise decreases in Q_tw,pot_ were significant in both groups before immobilization (IMMO −38.9%, d = 1.47; CG −36.1%, d = 1.35) and in the CG after immobilization (−34.9%, d = 1.91), but were smaller in the IMMO group after immobilization (−15.2%, d = 0.77). For potentiated twitch force, the magnitude of exercise-induced peripheral fatigue was reduced after the disuse period. Because no corresponding interaction was detected for M-wave amplitude or RMS/Mmax (LH M-wave: IMMO post-immobilization d = 0.34, p = 0.93; RMS/Mmax: d = 0.30, p = 0.15), the data provide no evidence that the attenuation arose from altered sarcolemmal excitability or propagation; processes beyond the muscle action potential, such as excitation–contraction coupling or the contractile apparatus, may be involved, although they were not assessed directly. Even so, this effect cannot be separated from the relative-intensity design: the lower MVC after immobilization reduced the absolute force sustained during the task and, with it, the metabolic and ischemic stress that drives peripheral fatigue. Comparable increases in endurance time during low-force sustained contractions after immobilization have been reported despite reductions in muscle size and strength (Yue et al., 1997; Semmler et al., 2000) and have been attributed to altered excitation–contraction processes, changes in activation strategy, and the lower absolute force linked to reduced MVC (Semmler et al., 2000; Clark et al., 2008). In the present study, the attenuated decline in Qtw,pot is best read as a smaller peripheral fatigue response to a lower absolute demand rather than as a true gain in contractile fatigue resistance. Post-exercise decreases in voluntary activation (VA) were significant in both groups before immobilization (IMMO −5.5%, d = 1.03; CG −6.4%, d = 1.18) and in the CG after immobilization (−5.7%, d = 1.32), but not in the IMMO group after immobilization (−2.0%, d = 0.38, p = 0.14). Notably, pre-exercise VA was already lower in the IMMO group after immobilization (81.03 ± 4.37%), which may have limited the scope for a further exercise-induced decline. Central fatigue is thought to involve interacting neural and muscular processes, including altered corticospinal excitability, changes in motoneuron properties and inhibitory feedback from group III/IV muscle afferents (Taylor et al., 2016). However, because no significant group × condition × exercise interaction was found for VA (p = 0.130), these within-condition differences should be read with caution and do not establish that short-term immobilization altered central fatigue mechanisms. Overall, short-term immobilization affected maximal force (MVC) and submaximal performance (Tlim) in different directions, and the exercise-induced decline in potentiated twitch force was attenuated afterward, as shown by a significant interaction (IMMO: −38.9% pre- vs −15.2% post-immobilization). No corresponding interaction was detected for VA, RMS/Mmax or M-wave amplitude, although the study was powered to detect only large interaction effects; differential changes in central drive or membrane excitability therefore cannot be excluded. Taken together with the relative-intensity design, the longer endurance time and the smaller peripheral fatigue response after immobilization are most plausibly explained by the lower absolute force demanded during the task rather than by an intrinsic adaptation of fatigue-related mechanisms.

A further consideration is that the post-exercise neuromuscular measurements were obtained after tasks differing not only in absolute force but also in duration. Following immobilization, participants sustained a lower absolute force for a longer period. Therefore, the pre- and post-immobilization assessments cannot be considered physiologically workload-matched, and differences in the post-exercise neuromuscular responses cannot be attributed exclusively to intrinsic changes in central or peripheral fatigability.

### Methodological considerations

Several limitations should be considered when interpreting the present findings. First, the relatively small sample limits statistical precision and prevented meaningful subgroup analyses according to sex or sport. Moreover, the inclusion of both male and female athletes from handball, basketball, and volleyball introduced biological and sport-specific heterogeneity that could not be examined within the present sample. Second, adherence to the immobilization protocol was based on participant records, and physical activity during the intervention period was not objectively monitored using accelerometry or another activity-monitoring method. Third, upper-arm MBA was estimated anthropometrically and therefore cannot be considered a direct measure of muscle morphology or muscle atrophy. Fourth, no *a priori* sample-size calculation was performed; the sensitivity analysis indicates that only large interaction effects (ηp² ≥ 0.26) could be detected with 80% power, so the non-significant interactions for VA, RMS/Mmax and M-wave amplitude should not be interpreted as evidence of no change. Fifth, participants and the investigators who conducted testing were not blinded to group allocation, and verbal encouragement during the endurance task may have introduced performance bias. In addition, sleep, nutrition, hydration, caffeine intake were not formally standardized before testing and may have contributed to between-session variability. Most importantly, the fatigue task was prescribed at 50% of the MVC measured during each visit. Because MVC decreased following immobilization, the post-immobilization task was performed at a lower absolute force and for a longer duration. Consequently, the neuromuscular assessments were performed after different mechanical and physiological exposures, and the smaller post-exercise reductions in MVC and Qtw,pot cannot be separated from differences in task demand. Future studies should incorporate complementary absolute-load- or work-matched conditions, objective activity monitoring, and direct imaging methods such as ultrasonography or magnetic resonance imaging.

## Conclusion

This study illustrates the dual effects of immobilization and exercise on neuromuscular function. Three weeks of arm immobilization significantly reduced maximal voluntary contraction strength and anthropometrically estimated muscle-and-bone area, indicating impaired maximal force-generating capacity. Despite this loss of maximal force capacity, endurance time during sustained submaximal exercise performed at the same relative, but a lower absolute, intensity increased after immobilization, and the exercise-induced declines in both maximal voluntary contraction and potentiated twitch force were attenuated.

No significant interaction was detected for voluntary activation or M-wave responses; however, given the sample size, differential changes in central drive or neuromuscular propagation cannot be excluded. Because the task was performed at a relative intensity, the smaller peripheral fatigue response and the longer endurance time after immobilization are best explained by the lower absolute force generated during the task rather than by a true gain in fatigue resistance. From a practical standpoint, these findings suggest that, after short-term immobilization, a longer endurance time at a given relative intensity should not be interpreted as preserved or improved fatigue resistance, because it is achieved at a lower absolute force. Whether these observations have implications for rehabilitation or progressive reloading was not tested and should be regarded as a hypothesis for future studies that include absolute-load-matched tasks and rehabilitation outcomes. Future studies are warranted to clarify the mechanisms underlying these adaptations and their implications for restoring neuromuscular function after limb immobilization.

## Data Availability

The raw data supporting the conclusions of this article will be made available by the authors, without undue reservation.
